# A retrospective review of 146 active and passive fixation bradycardia lead implantations in 74 dogs undergoing pacemaker implantation in a research setting of short term duration

**DOI:** 10.1186/s12917-018-1431-2

**Published:** 2018-03-27

**Authors:** Lynne E. Swanson, Barbara A. Huibregtse, Brian A. Scansen

**Affiliations:** 1 Research and Technology Center (Swanson), Boston Scientific Corporation, 4100 Hamline Ave North, Saint Paul, MN 55112 USA; 2 Preclinical Sciences (Huibregtse), Boston Scientific Corporation, 100 Boston Scientific Way, Marlborough, MA 01752 USA; 3 Department of Clinical Sciences (Scansen), Colorado State University, 1678 Campus Delivery, Fort Collins, CO 80523-1678 USA

**Keywords:** Canine, pacing, transvenous leads, complications

## Abstract

**Abstract:**

**Background:**

Canine veterinary patients increasingly benefit from implantation of transvenous pacemakers for bradyarrhythmias. No published data exist examining procedural outcomes of pacemaker implantation performed in the preclinical laboratory. The purpose was to review short term complication, infection, dislodgement, penetration rates, plus overall morbidity following pacemaker implantation in the research setting. A retrospective review of 74 Class A purpose-bred mongrels implanted with active (*n* = 89) and passive fixation (*n* = 57) intracardiac leads for dual (*n* = 72) or single (*n* = 2) chamber pacing was performed.

**Results:**

All leads were implanted successfully, meeting electrical implant criteria. Follow-ups typically occurred every 7 days (first month), then at 30 day intervals. Seroma formation was 1.4% and 10.8% at the venotomy and pulse generator site respectively. Overall infection rate was 1.4%. Overall dislodgement rate was 2.1%, (2 passive atrial leads, 1 passive ventricular lead). Overall fractures and insulation defects were zero. Two helix penetrations were noted incidentally post mortem, one at the right atrial appendage and one at the right ventricle (64 dogs, 128 leads evaluated), a 1.6% event rate. Major in-life adverse events were 5.4% (4 of 74 dogs), including 1 infection and 3 lead dislodgements.

**Conclusions:**

This review demonstrates a low complication rate with bradycardia lead implants in the short term (up to 180 days), in a high volume research setting. Lead type, implant technique, surgeon experience, healthy patient population, patient size and follow-up care play a role. This review also suggests active fixation leads in the right atrial appendage of dogs are safe and reliable.

**Electronic supplementary material:**

The online version of this article (10.1186/s12917-018-1431-2) contains supplementary material, which is available to authorized users.

## Introduction

Pacemaker and lead implantation procedures have been performed within the veterinary field for over 40 years, with the first implant in 1967 via a thoracotomy and epicardial placement of 2 leads for fixed rate pacing of 70 bpm [1]. Most veterinary patients undergoing pacemaker implantation today receive transvenous lead implants, resulting in a minimally-invasive procedure [2–4, 9, 14]. Advances in technology by the medical device industry have resulted in a wide range of active and passive transvenous pacing leads for human use that the veterinary clinician can now choose from for their specific patients, with refined smaller profiles, flexible lead bodies, different fixation types and insulation materials. Advances have also been made with pulse generators (PG), now smaller in size, with longer battery lives, offering a variety of software choices and device features for diagnostic and therapeutic programming. Such advances can pave the way for how the veterinary field manages their own patients in need of this therapy. Lower profile leads and smaller PGs allow for application of transvenous pacing to very small canine patients, who in the past may have had to undergo surgical placement of epicardial leads, but may now undergo a minimally invasive procedure. In tandem with these advances, implantation techniques have been refined in the preclinical setting with the ability to implant one, two, or even up to three leads in canine patients via a minimally invasive approach. All prototypes and iterations of pacing technology begin in the preclinical research realm, so the sharing of best research practices with the clinical veterinary community regarding lead types, implant techniques, and lead performance in terms of acceptable electrical criteria and device programming may facilitate transfer of knowledge for the betterment of veterinary clinical practice and veterinary patients.

Similar to human use, veterinary patients with symptomatic bradycardia, variations of atrioventricular (AV) block and sinus node dysfunction (SND) benefit from this device therapy. Breeds including west highland white terriers, miniature schnauzers and cocker spaniels tend to be predisposed to SND, while breeds such as Labrador retrievers and German shepherds appear predisposed to AV block [2, 3].

Published data have documented the clinical complications from lead implants exhibited in these veterinary patient populations. Overall rates for major complications consisting of lead dislodgement, infection, cardiac arrhythmias leading to death, battery issues, or programming errors range from 9 to 33% [3–15]. There are also clinical cases of cranial or caudal vena caval syndrome related to transvenous pacemaker implantation [16–20]. No published data exist, however, examining the procedural and post-operative outcomes expected in the short term, under ideal settings, in a healthy patient population, within a preclinical laboratory environment. Data on pacemaker complications in such an idealized setting may provide a useful comparison for future clinical veterinary studies. Therefore, the aim of this study was to characterize the canine population, lead types, surgical procedures, peri- and post-operative processes, in-life events and gross pathology in a research laboratory with several decades of experience implanting transvenous pacemakers in dogs.

## Materials and methods

All studies were reviewed by our Institutional Animal Care and Use Committee, with full approval and consent of all Class A research animals in accordance with the polices and guidelines of our institution, which is an AAALAC International accredited facility. All studies complied with animal use regulations as set forth in the United States Department of Agriculture Animal Welfare Act, 9, CFR, and adhered to the principles outlined in the "Guide for the Care and Use of Laboratory Animals," from the National Research Council.

Data were collected retrospectively from 71 dogs, from 2005 through 2016 with study durations of 42 to 180 days (29.6% 42–90 days and 70.4% 162–180 days) from complete data sets that were audited for FDA submission. An additional 3 dogs, that had implant durations of over 5 years (*n* = 3), were included after reviewing electronic animal records, written documentation, radiographs and other electronic media archived within 2 additional study protocols. A total of 2 single chamber device systems and 72 dual chamber device systems were implanted. The inclusion of animals from a study was based on specific lead construction type and PG device implanted. All 146 leads evaluated were bradycardia leads and the majority (61.0%, 89 leads) were of the active fixation helix lead type, of which 22.5% (20 leads) were open helix type and 77.5% (69 leads) were extendable/retractable type. The remaining implants consisted of passive leads with silicone tine fixation (39.0%, 57 leads). Animals were chosen for this review based on the use of lead types and PG devices comparable to those implanted clinically in veterinary patients. Implantable cardioverter-defibrillator and cardiac resynchronization devices were excluded. The lead models were 6 Fr to 7Fr bipolar silicone or polyurethane endocardial pacing leads for use in the right atrium (RA) or right ventricle (RV). The passive models had silicone rubber tines with a silicone rubber collar at the distal tip containing 1.0 mg dexamethasone acetate to reduce pacing thresholds after acute implantation injury.^1^ The active fixation models consisted of an open cork-screw helix coated with a mannitol tip^2^ or an extendable-retractable helix allowing for mapping capability prior to fixation with radiopaque markers allowing fluoroscopic visualization of full extension.^3^

### Inclusion criteria

All dogs were purpose-bred mongrel dogs and had been evaluated prior to approval and enrollment into any study. This pre-screening evaluation consisted of a physical exam, clinical pathology, heartworm status, ECG analysis and parasite screen. To qualify for inclusion, the dogs had to exhibit no external signs of health issues, normal blood parameters, negative heartworm status, negative result on fecal floatation and normal cardiac rhythm and morphology used to screen for chamber enlargement, conduction disturbances, arrhythmias, abnormal repolarization/depolarization changes and signs of ischemia. Once all criteria were met**,** dogs were deemed healthy and considered to be acceptable for enrollment into a study.

### Statistics

Statistical comparisons were made between active fixation and passive fixation leads for the rate of dislodgment and the rate of perforation for both the RA and RV using commercially available software.^4^ The Fisher’s exact test was used for statistical comparisons between groups. A *P* value < 0.05 was considered significant.

### Preparation and anesthesia

All dogs were fasted a minimum of 12 h prior to surgery and were bathed with a medicated shampoo^5^ within 24 h prior to implant.

Because of the retrospective nature of this review, there were slight alterations of anesthetic regimens and follow-up intervals. In general, each dog was sedated with 10 mg of IM butorphanol. Preventative antibiotics were given approximately 30 min prior to any incision and consisted of cefazolin^6^ (~ 30 mg/kg), given IV at the induction of general anesthesia. General anesthesia was induced with 200 mg of IV ketamine and 10 mg IV diazepam or 1 to 6 mg/kg of IV propofol. A surgical plane of anesthesia was maintained using isoflurane gas. After induction, the entire dorsal, lateral and ventral neck region encompassing the right side from the base of the head to the cranial edge of the scapulae was clipped and aseptically prepared for surgery. During implant, analgesia was maintained throughout the procedure with 10 mg of IV butorphanol every 1 to 1.5 h until the surgical portion was complete.

All dogs were placed on a ventilator with rate and tidal volume adjusted as needed to maintain stable physiological parameters. During the procedure, IV fluids (~ 5 ml/kg/h) with the vasopressor phenylephrine at a constant rate infusion (2–10 μg/kg/min) were commonly infused to maintain intravascular pressure and reduce total administered fluid volume.

All transvenous lead implants were performed by one of four dedicated veterinary interventionalists within the research facility with extensive experience in lead implantation. Each interventionalist was specifically trained on lead handling techniques and pacemaker implantation in dogs, pigs and sheep. Appropriate implant location was confirmed by meeting specific electrical criteria at implant, followed by appropriate pacing and sensing during the lead maturation period (typically the first 30 days), and subsequently, throughout the entire study duration. Full details of the implant procedures and post-implant animal care can be found in Additional file 1.

## Results

Outcomes were based on short term implantation of device systems ranging from approximately 2–6 months in duration, in an ideal environment, with dogs free of morbid cardiovascular conditions or other comorbidities. Class A mongrel dogs ranged in age from 5 to 27 months and in weight from 20.0 to 28.0 kg at time of implant. Data from 74 dogs and 146 lead implants were collected. Of the 146 leads evaluated, 28 were passive and 44 were active fixation leads implanted into the RA; 29 were passive and 45 were active fixation leads implanted into the RV. Of the 44 active fixation leads in the RA, 22.7% (10 leads) consisted of open helix leads and 77.3% (34 leads) were extendable/retractable helix lead types. Similarly, of the 45 active fixation leads in the RV, 22.2% (10 leads) were open helix leads and 77.7% (35 leads) were the extendable/retractable helix lead types.

In recovery, there were no adverse events recorded in any dog. Post-operatively, there were 3 dislodgements, all passive fixation leads (2.1% of all implants). Two preformed passive J leads dislodgements from the RA were noted on day 4 radiographs and 1 passive straight lead dislodgement from the RV was noted on day 7 radiographs (Table 1).

**Table 1:** Table of lead dislodgement and lead penetration findings

	Right Atrium			Right Ventricle		
Occurrence	Active Lead	Passive Lead	P value (Fisher’s Exact Test^b^)	Active Lead	Passive Lead	P value (Fisher’s Exact Test^b^)
Implanted	44	28		45	29	
Dislodged	0	2*	0.148	0	1^a^	0.392
Penetrations	1	0	1.000	1	0	1.000

^a^All dislodgements were passive fixation (preformed J in the RA; straight passive in RV)

^b^A *P* value < 0.05 was considered significant; however, the study was not powered for this endpoint

Overall infection rate was 1.4% (1 dog) across all 74 dogs. This dog exhibited seromas involving both the device pocket and venotomy site, with intermittent drainage and positive growth of staphylococcus on culture of the blood and site. Due to the short 90 day duration of implant for the study protocol this dog was assigned to, because the dog was healthy, and to avoid the risk of additional anesthesia and device explantation, the dog was maintained on antibiotics and anti-inflammatories throughout study duration. The dog remained bright, alert and active throughout the 90 day study period.

None of the studies reported any lead fracture, either during the in-life portion or upon examination post mortem. Electrical performance and function of all lead implants were deemed acceptable and fell within the accepted criteria at implant in unipolar and/or bipolar configurations (Table 2). Subsequently, leads did not exhibit any failure to sense or pace during the full in-life period, with the exception of the three dislodgements.

**Table 2:** Implant acceptance criteria

Parameters	Atrium	Right Ventricle
Threshold @ -0.5 ms PW	≤ 1.5 V	≤ 1.0 V
Sensing (amplitude)	≥ 2.0 mV	≥ 5.0 mV
Impedance (2.5 V or 5 V, 0.6 ms)	300–1800 Ω	300–1800 Ω

Excluding lead dislodgements and the infection noted above, there were no major adverse events, either during a follow up procedure or noted clinically during the in-life period in any of the 74 dogs. A major adverse event was defined as any in-life device related death, lead perforation or fracture, clinical event requiring life-saving therapeutic intervention such as pneumothorax, or any incident requiring additional surgery. A follow up was defined as a procedure that required sedation for radiographs or fluoroscopic imaging to monitor chronic lead fixation positioning as well as collection of electrical data with a programmer to measure unipolar and/or bipolar lead performance in specific pacing modes and programmable parameters; follow up events also included sedation plus anesthesia in order to perform extensive data collection on the test system performance, such as hemodynamic and electrical data before, during and after an MRI scan.

**Fig. 1:**
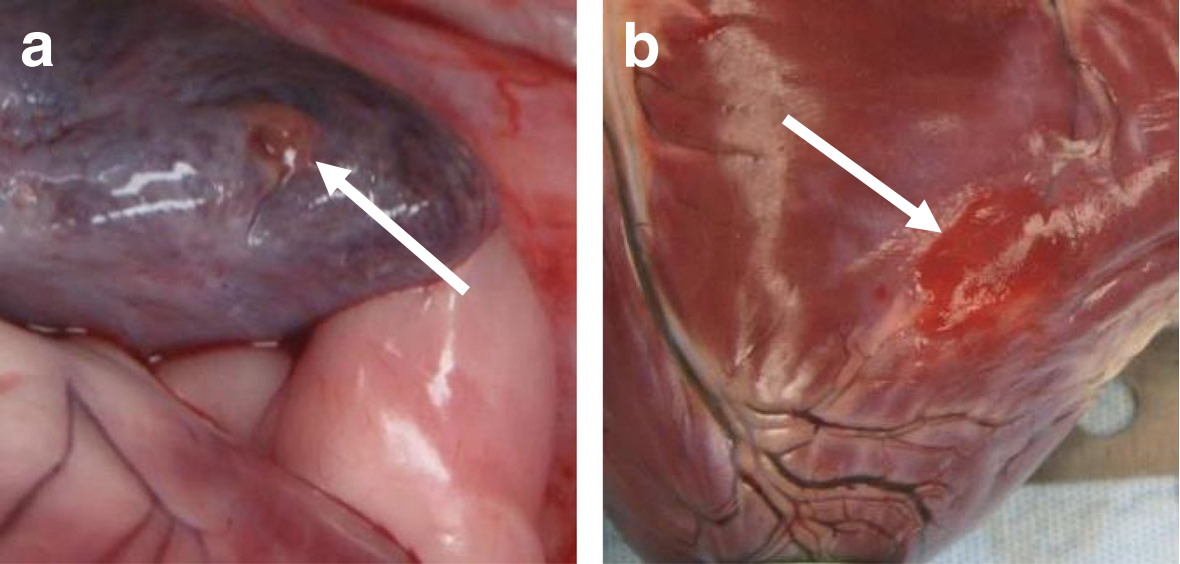
Typical gross presentation of RA appendage (**a**) and RV (**b**) lead helix penetration

**Fig. 2:**
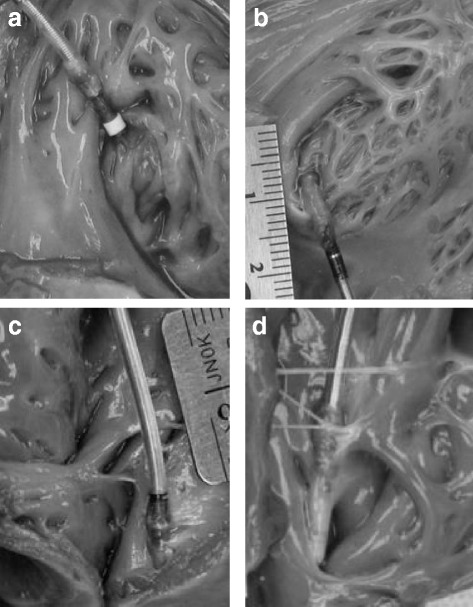
Variations on chronic mature endocardial lead implant sites. **a** RA appendage, active fixation lead, 90 days post implant; (**b**) RA appendage, active fixation lead, 47 days post implant; (**c** & **d**) Both right ventricles, active fixation leads, 180 days post implant

A post mortem necropsy was performed on 65 of the 74 dogs reviewed (3 still alive; 6 transferred to a separate study), consisting of 130 lead sites. Of the 130 implant sites, there were 2 helix penetrations of the epicardium observed, one active fixation extendable/retractable lead type in the RA and one active fixation extendable/retractable lead type in the RV, a 1.5% event rate (Fig. 1). These were incidental findings at necropsy only, the dogs not having exhibited any clinical signs indicative of a possible lead perforation (i.e. pneumothorax, pericardial effusion, focal lung lobe radiopacity, mediastinal changes etc.) either clinically or on serial radiographs and bloodwork. Variable encapsulation of the distal portion of the lead body was observed, as commonly noted with long term implants (Fig. 2). There were no other abnormal findings on the remaining 65 dogs at the RA or RV lead/tissue interface.

## Discussion

To the authors’ knowledge, this is the first time data has been collectively analyzed from a retrospective examination of pacemaker lead implantation performed in the preclinical laboratory setting. It should be emphasized that the preclinical research environment is well controlled, and studies are performed to show safety, not efficacy, for the regulatory agencies. Overall outcomes are based on evaluations in the short term, in overtly healthy patients. Complication rates in this review are therefore not directly comparable to real life clinical studies, due to the very different patient populations and co-morbidities found in clinical veterinary patients. However, the data may prove useful to veterinary cardiologists as baseline expectations for short term complications in near perfect conditions with young, healthy patients. Clinical management practices learned in the research laboratory may have applicability to practitioners who implant pacemakers in clinical canine patients. With the caveats noted above in mind, we did find some differences in complication rates between the data acquired in the study as compared to what has been documented in the literature gathered from the clinical canine setting. The reported rate of lead dislodgement in clinical patients varies from 6% [4] to 10% [7], with a value of 2.1% reported here. Reported infection rates for clinical veterinary patients vary from 1% [3, 14] to 6% [4], with 1.3% reported here. Reporting of the total number of major complications varies between studies, but includes reports of 9% of 104 dogs [14], 10% of 105 dogs [3], 23% of 136 dogs [7], and 27% of 33 dogs [4]; the total major in-life complication rate reported here was 5.4% for 74 dogs (1 infection, 2 RA appendage dislodgements, and 1 RV lead dislodgement).

Dogs are the model of choice for this type of preclinical research because of similar cardiac structure, function and size to that of humans. Much of the prior knowledge and experience related to bradycardia, tachycardia and pacemaker devices have been carried out using the in vivo canine model and this model is well characterized and well accepted by the FDA. Swine are a less useful model due to their growth dynamics and the historical observations that swine react differently to subcutaneous and sub muscular device implants, with a high incidence of abnormal reaction to foreign bodies, resulting in PGs eroding through the skin. There are several factors that may explain the lower rate of complications in this review. All study protocols for transvenous lead systems for this device company were implanted in larger (> 18 kg), USDA Class A origin, with little to no variability. This population inherently decreases the risk of inadequate vessel or chamber size for implantation and allows for appropriate lead slack, a more difficult procedure in smaller dogs in the real world setting. These research animals have no pre-existing disease and veterinarians or surgical research specialists working within a cardiovascular medical device company have the luxury of gaining considerable experience in lead implantation by sheer numbers alone, as that is the focus of much of the business development and innovation. Because of this, refinement of technique in lead site selection, tunneling, lead slack optimization, suture sleeve positioning and tightening, device pocket sizing and securement and being able to troubleshoot with electrical data challenges is easily achievable. Although nearly all the implants in this review were dual chamber device systems, which have comparable complication rates to single chambered systems in the veterinary field [4, 6], together, lack of patient morbidity, short to medium-term duration of implant, and operator experience likely play a large role in the incidence of complications, which is similar to what has been documented in human medicine [21]. Lastly, these animals are not discharged, but closely monitored on a daily basis, with highly technical human resources playing a big role in their aftercare. These animals are in a very well controlled environment, with close supervision on exercise restriction, daily observation of incision sites and daily bandage changes to insure constant pressure at the device pocket over a 2 week period, all which aid in limiting complication rates.

**Fig. 3:**
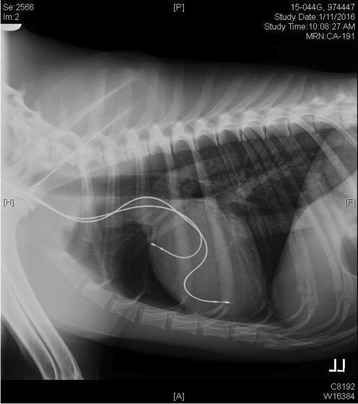
Standard slack allowance for RA and RV lead implants

Descriptions of these procedures and processes, acquired from decades of research on preclinical canine pacemaker implantation, to share with the clinical veterinary cardiology community are provided in Additional file 1. Lead slack redundancy appears to be the most common difference between preclinical techniques and reports from the clinical veterinary field. In human medicine, and in images from clinical veterinary reports [3, 14], lead bodies positioned in either the atrium or ventricle are implanted with little slack, which is considered adequate. This is logical for humans due to the bipedal stance and passive disposition of a typical cardiac implant patient. For dogs however, adequate slack is defined differently, with a gentle “S” curve (Fig. 3) required for the ventricular lead, to mitigate the quadruped stance, the changing dynamics, physics and length of the neck, and their spirited disposition. Too little slack, visualized as a straight line out the tricuspid valve, increases the risk of dislodgement once the dog is awake because of the lack of accommodation for neck and body movement. For the atrial lead, there should be enough slack such that the lead body rests just above the tricuspid valve annulus (Fig. 3). Too little slack, or an “L” shape, can result in too much tension on the helix and increases the risk of dislodgement. Too much slack in the lead body impinges on the tricuspid valve and could also result in excessive force against the helix tissue interface and increase the risk of myocardial penetration. Of potential rare complications that can occur, asymptomatic perforation is a known phenomenon and in most cases does not result in electrophysiologic consequences [22]. Passive lead perforation was not noted in this review, but has been documented clinically 7 weeks after implantation into the RV apex [23]. Implementing a small change in slack can result in large reductions in lead dislodgements. Although this is likely much more challenging due to smaller chamber sizes in the smaller patients that are a significant proportion of the patient population seen clinically in veterinary cases, slack initiatives should be implemented to help limit dislodgement.

An understanding of lead type construction and fixation properties is also important. This review demonstrated that both active and passive lead types appear safe and reliable for implantation in either chamber. There was no statistical difference between the two groups (active vs. passive) in the incidence of dislodgement or perforation (Table 1); however, the study was not powered for this endpoint.

This review also suggests that an active fixation lead implanted into the RA appendage (44 implants in this review) by experienced operators using appropriate techniques is safe in the short term and effective (in terms of lead electrical performance). Such an approach has not been fully utilized clinically, due to the perception that an optimal lead design for veterinary patients does not yet exist, that the thin walled RA appendage will not necessarily allow for proper fixation into the tissue, the presumption that there is a greater risk of perforation, and recognition of the need to develop optimized techniques to make atrial pacing more reliable [5, 24].

## Conclusions

Device safety is mandated by regulatory agencies and the research setting plays an important role assuring that these devices and the procedures associated with their implantation are safe and translatable in improving human health. Therefore, many studies in an animal model are performed to confirm safety with the advent of improved technologies resulting in refined leads and pulse generators. However, it is understood that a systemic review of relevant research studies in this setting does not exhibit the same diverse population, nor requirements of long term efficacy encountered in real life for practicing veterinarians. Interpretation of these results needs to be taken in context, understanding they are from data of short duration, in healthy patients, with little heterogeneity. With the above caveat, this review demonstrates a low complication rate with bradycardia lead implants in the short term (up to 180 days), in a high volume research setting. This review also suggests active fixation leads in the right atrial appendage of dogs are safe and reliable.

### Limitations

A direct comparison to clinical results in the veterinary field is limited in that these preclinical studies are short term in nature, with implants into overtly healthy patients. These animals are housed and cared for in a controlled environment with daily professional care for the full duration of implant. In addition, the volume of implants performed in a medical device company research facility is presumably higher than in the real life clinical setting, resulting in greater operator experience. The dogs are young and deemed healthy upon enrollment into every study, without any competing morbid condition. The dogs are also of larger size, all > 18 kg. Cranial or caudal vena caval syndrome is not recognized in this patient population, likely due to the larger size of preclinical dogs and therefore this review does not provide a representative sampling of complication rate secondary to single or multiple lead implantation. Lastly, duration of implant is dictated by FDA guidelines for safety requirements and the necessity for device/tissue interface data; the presence of only 3 dogs with long term outcomes (> 2 years) in this study prevents an analysis of long term complication rates that may be expected in the preclinical setting. It should also be noted that the definition of seroma in this review varied in documentation from minimal to moderate in size, but all incidences were counted, which may have skewed the data to a higher event rate than what is reported in clinical cases. Electrical data was taken to insure optimal location and device contact with the myocardium at implant and lead performance was confirmed by testing appropriate behavior in terms of sensing and pacing over the duration of the study, but data was not collected to assess efficacy. Despite these limitations, this review provides data on the expected complication rate of pacemaker implantation in the dog under idealized circumstances and suggests that the placement of active fixation leads in the RA of dogs is both safe and reliable in the short term.

## Additional file

Additional file 1: Implantation Procedures and Follow-up Care. Didactic description of the procedures and processes for intracardiac atrial and ventricular lead implantation and follow-up care in the research setting. (ZIP 303 kb)

## Abbreviations

AV: Atrioventricular
CFR: Code of Federal Regulations
FDA: Food and Drug Administration
PG: Pulse generator
PW: Pulse width
RA: Right atrium/atrial
RAA: Right atrial appendage
RV: Right ventricle/ventricular
V: Volts

## Footnotes

1 FINELINE™ II Sterox Atrial-J and straight silicone and polyurethane passive fixation pacing leads, Guidant Corporation, St Paul, MN; INGEVITY™ passive fixation Atrial-J and straight polyurethane pacing leads, Boston Scientific, St. Paul, MN

2 FINELINE™ II Sterox EZ, silicone and polyurethane active fix pacing lead, Guidant Corporation, St. Paul, MN

3 INGEVITY™ pacing lead, Boston Scientific, St. Paul, MN; DEXTRUS™ pacing lead, Guidant Corporation, St. Paul, MN

4 Minitab (version 17.1.0), Minitab Inc., State College, Pennsylvania

5 PYOBEN® Medicated Shampoo, Virbac Corporation, Fort Worth, Texas

6 Cefazolin, WG Critical Care, LLC, Vernon Hill, Illinois; West Ward Pharmaceutical Corporation, Eatontown, New Jersey
