# Supplementary material for: A retrospective review of 146 active and passive fixation bradycardia lead implantations in 74 dogs undergoing pacemaker implantation in a research setting of short term duration
Source: BMC Vet Res. 2018 Mar 27;14:112. doi: 10.1186/s12917-018-1431-2 (PMC5870196; doi:10.1186/s12917-018-1431-2)
Supplement: Supplementary file 1 — Implantation Procedures and Follow-up Care. Didactic description of the procedures and processes for intracardiac atrial and ventricular lead implantation and follow-up care in the research setting. (ZIP 303 kb) [file 12917_2018_1431_MOESM1_ESM.zip › Appendix 1_March 2018.docx]

**Additional file 1: Implantation Procedures and Follow-up Care**

A right external jugular venotomy is performed and RV and/or RA leads are implanted under fluoroscopic guidance using either firm or soft stylets.

*Ventricular Lead Implantation*

The ventricular lead is positioned into the RV apex using one of two techniques:

1. The stylet is pulled out of the lead approximately 10-12 cm, allowing for the floppy distal end to contact the atrial wall or other atrial structure, such as the tricuspid annulus, crista terminalis, or coronary os, prolapsing the lead body through the tricuspid valve. The stylet is then advanced into the distal tip of the lead guiding it down to the apex of the ventricle.
2. A gentle curve of 70 to 90 degrees is formed at the distal end of the stylet, the stylet is inserted fully into the lead, and the lead is advanced down into the atrium, floating across the tricuspid valve. Once across the valve, the curved stylet is replaced with a straight stylet and the lead tip advanced down into the RV apex.

For passive leads with silicone tines as the fixation mechanism, the tip is positioned as deep (ventral and caudal) into the RV chamber as possible with the stylet fully inserted to the distal end of the lead. For both types of active fixation leads, the distal tip is rotated into position based upon the instructions for use. For the open helix lead type, the corkscrew helix is coated with a mannitol capsule that begins to dissolve once it enters the blood pool and requires approximately 5 minutes before full fixation can be attempted. Fixation into the myocardium prior to the mannitol disappearing can result in partial fixation and a concurrent increased risk of dislodgement. The lead body is rotated clockwise at the venotomy site delivering torque down to the tip, leaving the stylet stationary. For the extendable retractable lead type, fixation is accomplished with a fully inserted stylet, using clockwise turns with a rotational wrench positioned at the terminal pin. The number of turns is dependent on lead type, but turns are always counted and never exceed the maximum number of turns based on the instructions for use. Fixation into the myocardium (active fixation leads only) is confirmed by meeting 4 distinct endpoints:

1. With manual rotation of the lead tip, visual verification of counterclockwise release of the torque at the terminal end is accomplished by positioning the palm of one hand under the lead, releasing torque off the lead with the other hand, and allowing the lead body to “spin back” to normal. If spin back is not visualized, the lead is rotated counterclockwise to release the helix from the myocardium and re-implanted. If the extendable/retractable lead is implanted, confirmation of full extension is verified not by spin back, but via fluoroscopy based off of radiopaque extension markers.
2. Fluoroscopic verification that removal of the stylet does not change lead tip location
3. Fluoroscopic verification that advancing redundant lead slack into the chamber does not result in dislodgement.
4. Verification that appropriate electrical amplitude, impedance, and thresholds are met (**See Table 2 in main body of manuscript**).

If any one of these four criteria is not met, the lead is not considered to be fully fixed into position and requires repositioning.

*Atrial Lead Implantation*

Straight active fixation leads are the preferred lead type, implanted with a two-stylet technique. The lead is advanced under fluoroscopic guidance past the base of the heart and into the caudal vena cava with a straight stylet. The stylet is removed and a preformed J stylet inserted. As the “J” takes shape, the lead body is retracted up into the RA chamber. Using the hub of the stylet at the terminal end of the lead, the “J” is rotated cranio-dorsally into the RA appendage. Visualization of a windshield-like motion of the lead body on fluoroscopy is initial verification of appropriate placement within the appendage. The distal tip is positioned perpendicular to the endocardial wall at a 90 degree angle by “opening up the J” and pulling back on the lead body prior to fixing it into position. Once the helix is screwed into place (via manual clockwise rotation of the lead body or via wrench rotation and extension), the lead is tested for stability using the same 4 steps described above for ventricular lead implant.

*Positioning of Leads*

To ensure suitability of the implant site, impedance, voltage threshold, and sensing data are collected using a pacing system analyzer with alligator clips attached to the terminal lead pins prior to connecting the leads to the pulse generator (PG). If electrical data falls within acceptance criteria, the correct amount of lead slack for each lead is verified fluoroscopically and the leads secured into position with installed suture sleeves. All leads are anchored at the jugular venotomy site and underlying muscular fascia via individual suture sleeves at each suture sleeve groove (2 grooves per sleeve) typically using 2-0 Prolene. Umbilical tape, or alternatively Retract-O-Tape^g^, positioned 2-3cm distal to the venotomy site, is placed around the jugular vein and lead body and carefully tightened using a mosquito clamp, preventing the sleeve from slipping too deep into the vein. If 2 suture sleeves are being placed due to a two-lead implant, one sleeve is positioned in this manner, with the remaining sleeve positioned just cranial and adjacent to it, typically lying outside of the vein. Sutures are placed around both grooves to tighten the sleeve around the lead, with placement of an additional circumferential ligature into the muscle fascia below to anchor the lead. For proper slack, the ventricular leads demonstrate an “S” shape within the ventricular chamber and the atrial leads exhibit a deep “J” within the atrial chamber, with the base of the “J” just above the tricuspid valve (**See Figure 3 in main body of manuscript**).

*Pulse Generator Implantation*

A second incision is made dorsal to the venotomy site for placement of the PG. An undersized subcutaneous pocket is created via blunt dissection in a dorsal or dorsal-lateral cervical location. The lead(s) are tunneled to this site, inserted into the header of the PG, with the terminal tip of the lead visualized beyond the last set screw and set screws tightened. The wrench used to tighten these screws creates a clicking sound during clockwise rotation. Only 1 click is necessary to ensure contact and further clicks are unnecessary and may result in stripping the screw. Any port not in use is fitted with a port plug. The device is interrogated for the lead pacing threshold, pacing impedance and sensing of R and P waves. To insure proper contact and lead performance, 3 tests are performed to confirm set screws are tightened appropriately: (1) after insertion into the header, the distal portion of the terminal pin is visualized beyond the final set screw; (2) after tightening the set screw(s) with at least 1 click of the wrench, the lead body is grasped and pulled back gently without slippage out of the header (3) interrogation of the device yields normal pacing thresholds, sensing amplitudes, and impedances prior to closing the pocket. Any noise or abnormal electrical data should alert one to remove, clean off the terminal pin and set the screws again. Thresholds are set at 2x capture threshold to allow for an appropriate safety margin. The PG is placed either directly into the subcutaneous pocket in a subset of studies or into a sterile parsonnet polyester pouch^h^ prior to being placed into the subcutaneous pocket. This pouch is used to decrease the risk of seroma formation and twiddler’s syndrome, the coiling of lead bodies around the PG in the subcutaneous pocket resulting in lead dislodgement (**Additional file 1:** **Figure S1**). The device is secured to the underlying muscular tissue via the preplaced suture hole on the header (the portion of the PG that houses the terminal lead bodies with set screws) using 0 Prolene.

*Post-implantation Care*

All incisions are closed in a standard manner in 3 layers. An anti- microbial sealant^i^ is applied to the site and in later studies transparent dressing^j^ has been applied over both incisions. A fluoroscope image is taken to document the final positions of all leads in each dog.

After recovery, each dog has a cervical or cervical-thoracic pressure bandage placed encompassing the PG pocket and venotomy site. This bandage is removed, the incisions visualized, and a new bandage applied once a day for a minimum of 14 days or until there is full resolution of any seroma. Benadryl cream and/or baby powder is applied as needed for prevention of moist dermatitis. Exercise restrictions are implemented for 14 days.

Post implant, dogs are medicated with deracoxib (~3 to 4 mg/kg) PO q24h starting the evening of surgery and continuing for 3 to 5 days. Long acting cefovecin sodium (8 mg/kg IM) is the sole post-operative antibiotic administered, given once the evening after surgery. Dogs may receive a dose of acepromazine (0.05 mg IV) anxiolytic during recovery.

Follow-up procedures consist of radiographs in left lateral and ventral-dorsal positions as well as device interrogation for collection of electrical data and standard blood chemistries. Gentle restraint is employed during radiography and electrical data collection with the dog standing; alternatively, light sedation with xylazine (up to 1.1 mg/kg IV) is given for both procedures when electrical data are collected in left lateral position.

The timeline for follow-up has varied across studies, but in general involves standard rechecks on days 4, 7, 14, 21, 28, 60, 90, 120, 150, 180, and 1 year post-operatively with annual evaluations thereafter.

**Additional file 1 Endnotes**

^g^ Retract-O-Tape^®^ Quest Medical, Inc., Allen, TX

^h^Bard^®^ Parsonnet™ Pulse Generator Pouch, BARD Peripheral Vascular, Tempe, AZ

^i^Integuseal microbial sealant, Halyard Health, Alpharette, GA

^j^ Tegaderm, 3M Corporation, St. Paul, MN

**Additional file 1 Figures Legend**

Additional file 1: Figure S1. (A) Polyester Dacron pouch, cut open; (B) PG encased, both 90 days post implant.
