# Supplementary figures and images for: A retrospective review of 146 active and passive fixation bradycardia lead implantations in 74 dogs undergoing pacemaker implantation in a research setting of short term duration
Source: BMC Vet Res. 2018 Mar 27;14:112. doi: 10.1186/s12917-018-1431-2 (PMC5870196; doi:10.1186/s12917-018-1431-2)

## Slide 1
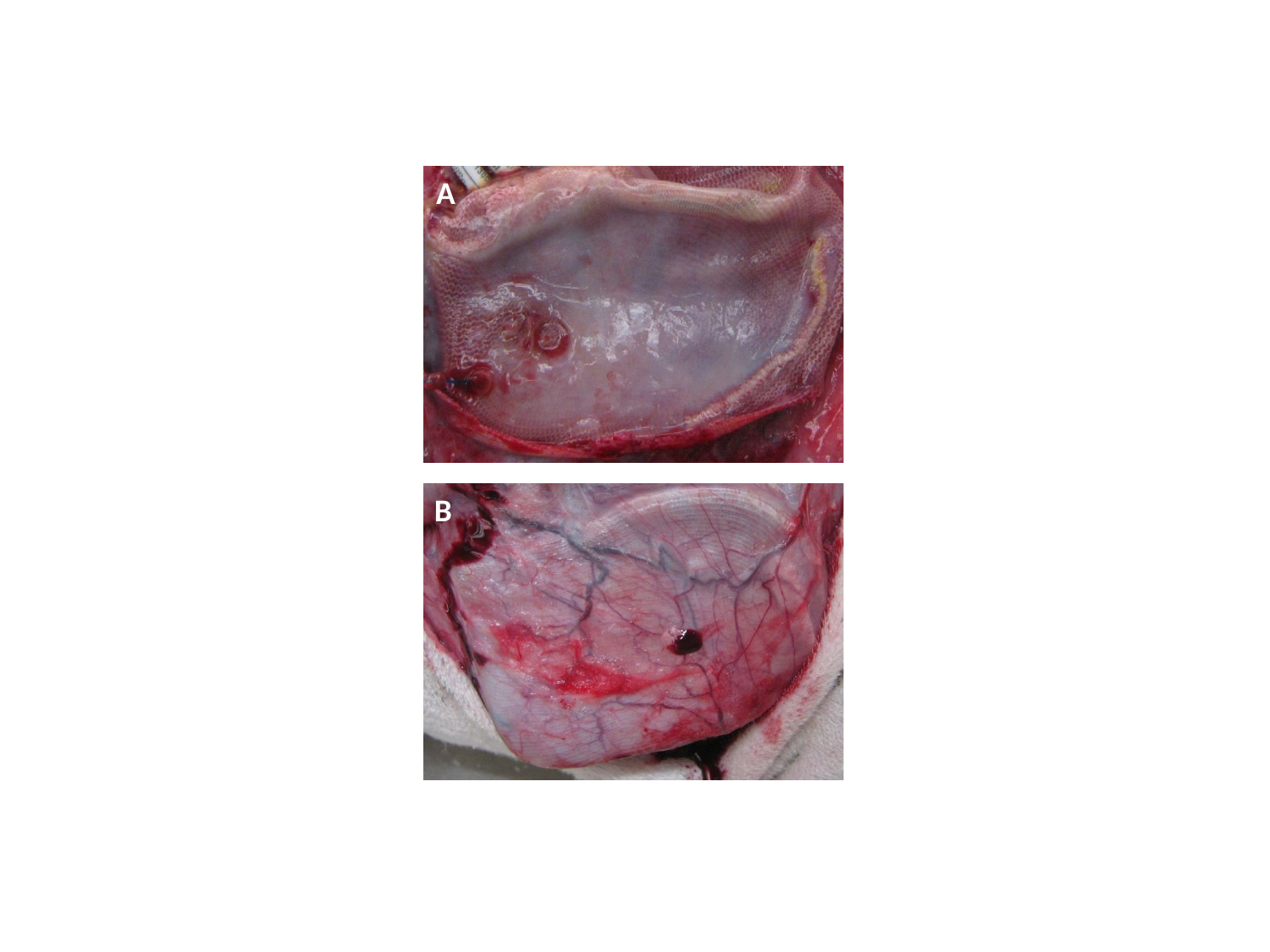

A
B

Supplement: Supplementary file 1 — Implantation Procedures and Follow-up Care. Didactic description of the procedures and processes for intracardiac atrial and ventricular lead implantation and follow-up care in the research setting. (ZIP 303 kb) [file 12917_2018_1431_MOESM1_ESM.zip › LSwansonAppFig1_Revised_Jan 2018.pptx]
